# Monotony

**Published:** 1887-03-19

**Authors:** 


NURSES' TRIALS—MONOTONY.

WEARINESS of mind and body is undoubtedly the unavoidable trial of a nurse's life; but is it not sometimes the outcome of monotony? How often, over and over again, a nurse has to listen to the same old complaints and wants of one set of patients after another, until, if she had not learned the truth of Christ's words, "Possess ye your souls in patience," she could scarcely check an impatient word sometimes. That is an unavoidable monotony; another is the necessary regular routine of daily work; and another, which is very often, but ought not to be, the monotony of off-duty time. If a nurse, as is often the case, comes to a strange town, how long she may be there, and yet never know *socially* anyone outside the hospital. Many people have an idea a nurse must be of different material from other women,—certainly at times she almost needs be; but there are times when her ordinary woman's nature longs for social intercourse outside and away from the hospital. That is not always easy, for a nurse's off-duty time is limited. But people who have time to spare and homes of their own, if they would only find out more about the individual nurses in their own town, and sometimes invite them to their homes; for oh! how often the nurse who has to listen to and feel for the sorrows of the sick under care—how often her own life and heart is sad within her—though she shows it not; for often it is because she, who had once a home, and has one no more, needs to be a nurse. Of course, nurses range widely in the social scale, but surely there are kindred spirits for all in the world somewhere. Some may say a great deal is now done for nurses. There certainly is, and in the large hospitals a nurse's is a bright, rather than a sad life, as is often thought.

But the monotony of the lives of those in small hospitals might easily be made less, if some people who want to do good to their fellow-creatures would get to know some nurse in their own town—know her as a woman, forget her for the time being as a nurse, and, above all, not talk hospital to her, but let her see and feel ordinary healthy outside social life. She would go back to her work stronger and better, so, through good outside influence, more good would come to the patients under that nurse's care. That is one way of relieving the monotony of a nurse's life.

